# Baidu Jieduan granules, traditional Chinese medicine, in the treatment of moderate coronavirus disease-2019 (COVID-19): study protocol for an open-label, randomized controlled clinical trial

**DOI:** 10.1186/s13063-021-05418-y

**Published:** 2021-07-22

**Authors:** Wen Zhang, Qin Xie, Xiaoming Xu, Shuting Sun, Tian Fan, Xinxin Wu, Yao Qu, Jinhua Che, Tingrong Huang, Huacheng Li, You Zheng, Chao Jiang, Bangjiang Fang, Shuang Zhou

**Affiliations:** 1 Department of Emergency, LongHua Hospital, Shanghai University of Traditional Chinese Medicine, 725 Wanping South Road, Xuhui District, Shanghai, 200032 NO China; 2 Wuhan Mental Health Center, Wuhan, 430012 China; 3 Medical Informatics Department, Wuhan Center Hospital, Tongji Medical College, Huazhong University of Science and Technology, Wuhan, 430014 China; 4 Clinical Medical College of TCM, Hubei University of Chinese Medicine, NO.1 Tanhualin, Wuchang District, Wuhan, 430065 Hubei China; 5 Medical Department of Hubei Minzu University, Enshi, 445000 Hubei China; 6 Huangshi Hospital of TCM (Infectious Disease Hospital), NO.6 Plaza Road, Huangshi Port District, Huangshi, 435000 Hubei China; 7 The third Department of Neurology, The Second Affiliated Hospital of Xi’an Medical University, NO.167, Textile City East Street, Baqiao District, Xi’an, 710032 Shanxi China; 8 Shanghai University of Traditional Chinese Medicine, 1200 Cai Lun Road, Zhangjiang Hi-Tech Park, Pudong New Area, Shanghai, 201203 China

**Keywords:** COVID-19, Baidu Jieduan granule, Efficacy and safety, Randomized controlled trial

## Abstract

**Background:**

Currently, coronavirus disease-2019 (COVID-19) is continuously and rapidly circulating, resulting in serious and extensive effects on human health. Due to the absence of antiviral medicine for COVID-19 thus far, there is a desperate need to develop effective medicine. Traditional Chinese medicine (TCM) has been widely applied in the treatment of epidemic diseases in China, with the aim of achieving clinical efficacy and decreasing the use of antibiotics and glucocorticoids. The aim of this study was to evaluate the efficacy and safety of Baidu Jieduan granules in treating COVID-19.

**Methods/design:**

This multicentre, open-label, randomized controlled trial will be conducted in 300 patients with COVID-19. The patients will be randomly (1:1) divided into a treatment group and a control group. All patients will receive standard therapy at the same time. Patients in the experimental group will receive Baidu Jieduan granule treatment twice a day for 14 days. The outcomes will be assessed at baseline and at 3, 5, 7 and 14 days after treatment initiation. The primary outcome will be the rate of symptom (fever, fatigue and coughing) recovery. Adverse events (AEs) will be monitored throughout the trial.

**Discussion:**

The study will provide high-quality clinical evidence to support the efficacy and safety of Baidu Jieduan granules in the treatment of moderate COVID-19, and enrich the theory and practice of TCM in treating COVID-19.

**Trial registration:**

Chinese Clinical Trial Registry ChiCTR2000029869. Registered on 15 February 2020

**Supplementary Information:**

The online version contains supplementary material available at 10.1186/s13063-021-05418-y.

## Introduction

An outbreak of coronavirus disease-2019 (COVID-19) in Wuhan city, China, has resulted in a global health emergency, with over 61.8 million confirmed cases, including over 1.4 million deaths as of 29 November 2020 around the world [1]. The number of patients may be underestimated due to asymptomatic individuals or individuals with moderate symptoms [2]. At present, scientists are sure that COVID-19 is caused by a new virus, named “severe acute respiratory syndrome coronavirus 2 (SARS-CoV-2)” [3]. Patients suffer from fever, fatigue and dry cough, with some progressing to breathing difficulties, acute respiratory distress syndrome or sepsis [4]. The disease is diagnosed by clinical characteristics, epidemiological history and laboratory test results according to the “Guidelines for the Diagnosis and Treatment of Novel Coronavirus (COVID-19) Infection by the National Health Commission (Trial Version 7)” [5].

Currently, there are no vaccines or specific antiviral drugs for COVID-19; meticulous supportive therapies are the cornerstone. Traditional Chinese medicine (TCM) has an extensive clinical history in preventing and treating epidemic diseases [6]. From the Ming Dynasty, “Wen Yi Lun”, a famous ancient medical book, mentions that epidemic diseases can be contagious to other people and transmitted through the respiratory tract and digestive tract. Modern medical practitioners have suggested that COVID-19 should be classified as damp-warm pestilence in TCM [7]. Dampness obstructs the Qi mechanism, leading to more dampness and warmth. Furthermore, damp warmth may also injure Yin and Qi and cause pathogenic toxicity and blood stasis. TCM has been widely applied to cure patients with COVID-19 associated with damp-warm syndrome, especially in combination with Western medicine, and can reduce the use of antibiotics and glucocorticoids [8]. Based on the above TCM pathogenesis of COVID-19, our team proposes “San Tong strategies” [9], integrating three kinds of strategies, including relief of the exterior syndrome, diarrhoea and diuresis, and the “truncation and reversion” strategy [9, 10], rationally adopting catharsis of the large intestine to prevent and treat sepsis and prevent and treat the disease in a timely manner. On the foundation of the San Tong strategies, we developed Baidu Jieduan granules, a formula consisting of *Rheum palmatum L.* stem (*Dahuang*), *Sargentodoxa cuneata (Oliv.) Rehd. et Wils.* (*Hongteng*), *Taraxacum mongolicumHand.-Mazz.* (*Pugongying*), *Raw Gypsum* (*Sheng Shigao*), Herba Ephedra (*Mahuang*), *Talcum* (*Huashi*), *Amygdalus Communis Vas* (*Xingren*), *Radix Glycyrrhizae* (*Gancao*), *Verbena officinalis L.* (*Mabiancao*), *Polygonum cuspidatum* (*Huzhang*), *Scutellariae Radix* (*Huangqin*) and *Bombyx batryticatus* (Jiangchan).

Baidu Jieduan granules evolved from the classical TCM prescription Maxing Shigan decoction and our experiential prescription, Jinhong decoction. Our previous studies have demonstrated that Jinhong decoction, composed of *Rheum palmatum L.* stem, *Sargentodoxa cuneata* and *Taraxacum mongolicum*, can inhibit the levels of TNF-α, IL-6, IL-8 and other inflammatory cytokines; protect against excessive inflammatory responses; and maintain an organism’s balance between inflammation and anti-inflammatory responses in infectious diseases [11, 12]. Recent research has shown that Maxing Shigan decoction is regarded as an antipyretic, anti-inflammatory, antiviral, antitussive and antiasthmatic agent and can be used to treat COVID-19 [13]. Therefore, Baidu Jieduan granules should have beneficial and curative effects on COVID-19. However, there is insufficient evidence to show the clinical efficacy of Baidu Jieduan granules. Hence, we aim to conduct a multicentre, randomized trial to evaluate the efficacy and safety of Baidu Jieduan granules in the treatment of COVID-19. Furthermore, this trial can serve as a reference for TCM in the prevention and treatment of COVID-19 worldwide.

## Methods/design

### Study design and settings

**Fig. 1:**
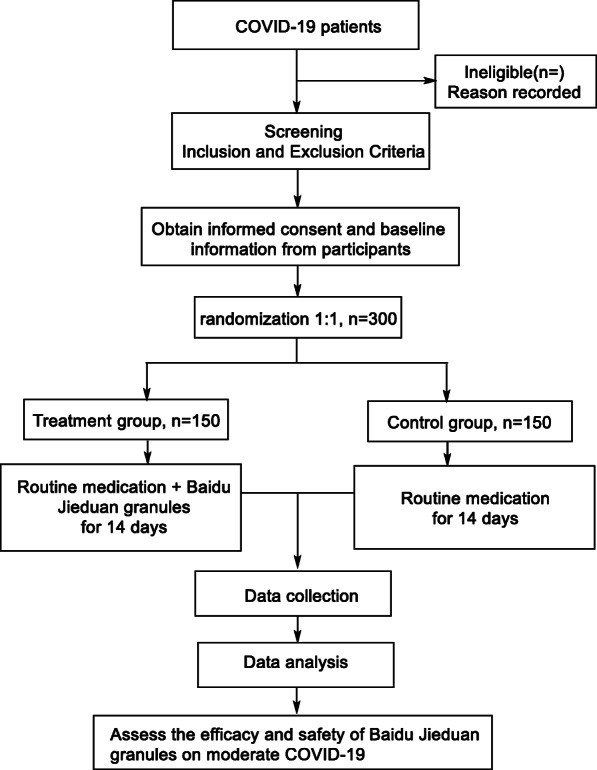
The flow chart of the efficacy and safety of Baidu Jieduan granules for moderate COVID-19. COVID-19: coronavirus disease-2019

The study is designed as a randomized, placebo-controlled, multicentre trial, which will be conducted at four medical centres selected by the expert committee: the Huangshi Hospital of Traditional Chinese Medicine; Tongji Hospital, Tongji Medical College, Huazhong University of Science and Technology; LaoHeKou Traditional Chinese Medicine Hospital; and Leishenshan Hospital of Wuhan. A total of 300 participants fulfilling the eligibility criteria will be randomized into two groups (Baidu Jieduan granule group and control group) at a ratio of 1:1. The study flowchart is illustrated in Fig. 1. The Standard Protocol Items Recommendations for Interventional Trials (SPIRIT) checklist is presented in Additional file 1.

### Population

Participants with COVID-19 will be recruited from 4 study sites in China. Patients who are eligible according to the inclusion criteria and who provide informed consent will be screened during the clinical trial period. Patients meeting any of the exclusion criteria will be excluded before randomization. Recruitment will last for 5 months, from February 2020 to December 2020.

### Inclusion criteria

Subjects must meet all of the following requirements:

1. Patients 18–85 years of age
2. Patients with COVID-19 confirmed by SARS-CoV-2 nucleic acid testing of respiratory specimens showing positive results
3. Patients providing written informed consent

### Exclusion criteria

The exclusion criteria are as follows:

1. Pregnancy and lactation
2. Severe primary diseases influencing survival, including malignant tumours, haemorrhagic disease and HIV
3. Severe liver and kidney dysfunction
4. Immunosuppressant therapy or an organ transplant within the previous 6 months
5. Participation in another clinical trial in the last 30 days
6. Allergy to one or more components in the herbal TCM prescription

### Patient withdrawal

1. Enrolment without meeting the inclusion criterion
2. Unsuitability for the trial due to deterioration or recovery of the patient’s condition
3. Poor patient compliance
4. Voluntary withdrawal

Upon patient withdrawal from the trial, all collected data up to the time of withdrawal will remain in the database. Safety-related information may be obtained until the 14th day after withdrawal if possible.

### Plan for retaining participants

Methods are needed to promote participant retention. First, we will select the coordinator and nurse carefully and train them strictly before the trial begins. Furthermore, we will focus on daily communication and monitoring. The coordinator and nurse should have empathy and good communication skills to gain the patient’s trust to improve patient retention and completion of the follow-up period. In addition, we will provide extra incentives.

### Sample size

A similar clinical trial has been conducted using TCM to decrease the rate of symptoms (fever, fatigue and coughing) in patients with COVID-19 [14]. The rate of recovery of clinical symptoms was 91.2% in patients who received TCM treatment and 61.1% in patients who received conventional treatments. Considering 90% power with a significance level of 10%, as well as a rate of withdrawal and loss to follow-up of 10% [15], we plan to recruit at least 150 participants in each group to achieve sufficient precision in a subsequent full trial.

### Randomization and blinding

The enrolled participants will be randomly assigned to the Baidu Jieduan granule group or the conventional treatment group according to random codes. The random sequence of 300 participants from 4 medical centres will be generated by SPSS 19.0 and stratified by centre. Two sets of random codes will be stored in opaque envelopes, which will be sent to the research sponsor and each of the centres. Thereafter, the drug administrators will arrange the nurse to dispense the study drug in the order of random numbers. This will be an open-label study. The statistical analysis will be carried out by the Professors of Statistics of Shanghai University of Traditional Chinese Medicine, who will be blinded to the patient allocation.

### Interventions

The Baidu Jieduan granules are composed of 15 g of *Rheum palmatum L.* stem (*Dahuang*), 30 g of *Sargentodoxa cuneata (Oliv.) Rehd. et Wils.* (*Hongteng*), 30 g of *Taraxacum mongolicumHand.-Mazz.* (*Pugongying*), 45 g of *Raw Gypsum* (*Sheng Shigao*), 9 g of Herba Ephedra (*Mahuang*), 45 g of *Talcum* (*Huashi*), 12 g of *Amygdalus Communis Vas* (*Xingren*), 9 g of *Radix Glycyrrhizae (Gancao)*, 30 g of *Verbena officinalis L.* (*Mabiancao*), 30 g of *Polygonum cuspidatum* (*Huzhang*), 30 g of *Scutellariae Radix* (*Huangqin*) and 12 g of *Bombyx batryticatus* (Jiangchan), packaged in two bags. The Baidu Jieduan granules will be administered orally two times a day for 14 days. The Baidu Jieduan granules are manufactured by Beijing Tcmages Pharmaceutical Co., Ltd. (number: Jing 20180032).

The participants will be categorized into two groups receiving either standard Western medicine alone according to the *Protocol for Diagnosis and Treatment of Novel Coronavirus Pneumonia* (7th edition) or the combination of Baidu Jieduan granules two times a day for 14 days plus standard care. Routine care includes early fluid resuscitation, antimicrobial anticoagulants, nutritional support and other treatments. Other TCM therapies, including TCM injections and other oral herbal medicines, will be prohibited.

### Outcomes and measurements

The primary outcome will be the proportion of negative SARS-CoV-2 nucleic acid results on the 14th day. The secondary outcomes will be the time to symptom recovery, the proportion of cases with reversion on chest CT, the time to negative SARS-CoV-2 nucleic acid results and the proportion of participants regrouped as severe cases. Symptom recovery is defined as the complete disappearance of fever, fatigue and coughing symptoms.

Evaluation of the primary and secondary outcomes will occur at five points (before treatment and the 3rd, 5th, 7th and 14th days of hospitalization).

### Safety outcomes

All safety-related indexes, including vital signs, complete routine blood indexes, routine general urine indexes, biochemical indexes, faecal occult blood test results and electrocardiogram results, will be tested and recorded in the CRF at every visit. The biochemical test includes C-reactive protein, hepatic function indicators (alanine transaminase, aspartate transaminase, alkaline phosphatase, total bilirubin and gamma-glutamyltransferase) and renal function indicators (blood urea nitrogen and serum creatinine). Any AE will be evaluated by a professional researcher at each visit and recorded on the CRF.

### Adverse event (AE) reporting

An AE is defined as any undesirable syndrome connected with the administration of Baidu Jieduan granules in a patient [16]. Any AE needs to be recorded in the form immediately and then evaluated by the physician and the corresponding coordinator. All related information, including the occurrence time, severity, duration, measures adopted and outcome, can be reported to the sponsors, ethics committees and drug regulatory authorities in accordance with the provisions. We will follow all subjects with AEs.

### Statistical analysis

We will not conduct subgroup or adjusted analyses of the trial. The independent statistical analysts will be responsible for the data analysis. All patients will be analysed on the basis of intent-to-treat analysis, including all randomized subjects who take Baidu Jieduan granules at least once and have follow-up information recorded. In the case of missing data, multiple imputation will be carried out to generate missing values. A per-protocol analysis, which includes patients who completed the trial without conflict with the major protocol, can be conducted as appropriate. Then, the statistician will submit statistical reports in a timely manner to the study director. The statistical analysis will be performed in a blinded manner using SPSS 20.0 software. Continuous variables characterizing each study group will be expressed as the mean with standard deviation or median with interquartile range. Categorical variables will be reported as frequencies and proportions. Continuous outcomes will be analysed by unpaired Student’s *t*-test or Wilcoxon’s nonparametric statistic, while count data will be compared using a chi-square test or Fisher’s exact test. All statistical tests will be two-sided. *P* < 0.05 will be considered statistically significant. We will not carry out an interim analysis.

### Trial oversight

#### Coordination Centre

The membership of the Coordination Centre will consist of clinical experts, statisticians and quality control experts from each centre. The centre will be responsible for the management of the clinical research trial, cooperative hospitals and preclinical trial training courses and resolving key issues in the whole process of study.

#### Quality control

All centres and researchers will be regularly monitored and supervised by a clinical research organization (CRO) throughout the trial, according to the standard protocol.

### Data management

**Fig. 2:**
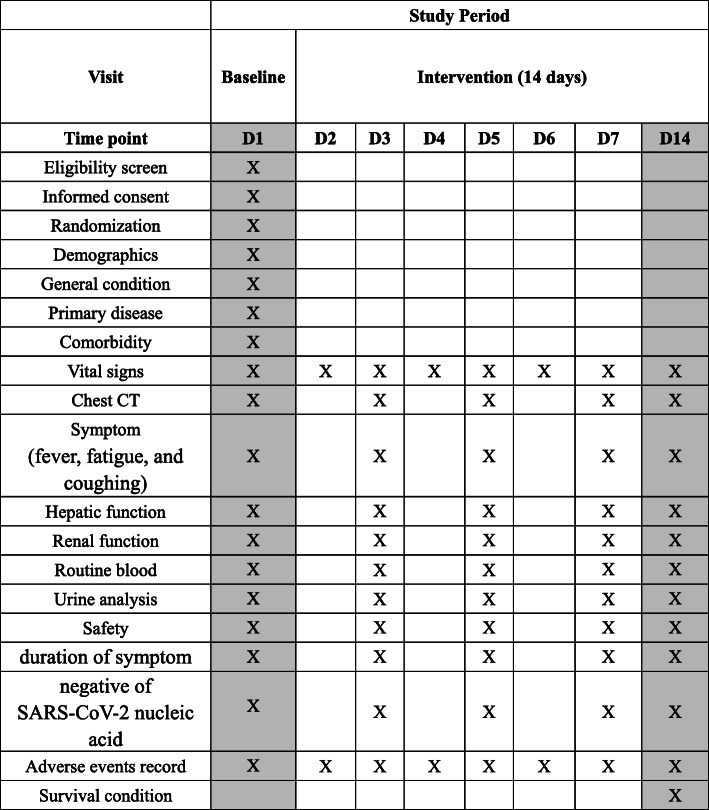
Study procedures and assessments. CT computed tomography, SARS-CoV-2 severe acute respiratory syndrome coronavirus 2

Trained research staff will collect trial data carefully according to a standard protocol and will complete paper case report forms (CRFs) accurately, completely, timely and reliably. The data will be entered into the electronic data capture (EDC) system and regularly reviewed by a clinical research associate (CRA). If any changes are implemented by researchers, feedback will be provided to the researchers and CRA. All modifications and paper CRF transfers between investigators, inspectors and data managers will be documented and maintained appropriately. The data administrators will lock the data on completion of the study. The medical information of all the study participants will be kept strictly confidential. The SPIRIT flowchart of the study is shown in Fig. 2. Research findings will be announced via publication and conferences, both nationally and internationally. After the study is published, the data will be made available in an appropriate manner.

### Quality control

All investigators of the trial will be trained strictly and comprehensively by the State Food and Drug Administration, following Good Clinical Practice (GCP), to thoroughly comprehend the procedure of the trial and improve compliance. All qualified researchers will collect data and complete the CRF in an accurate and complete manner. After discharge, the subjects will be followed up by phone. Regular monitoring and inspections will be conducted by the CRO once a month to confirm the reliability of the related research data and the trial process. The CRO will check details on informed consent, the inclusion and exclusion criteria, the original data, the management of AEs, the process of storage and distribution of the research drugs and the CRF.

### Ethics

This trial will comply with the principles of the Declaration of Helsinki and the regulations on the quality management of clinical trials in China. The research protocol has been approved by the Ethics Committee of Huangshi Hospital of Traditional Chinese Medicine (approval number HSZY-PJ-2020-002-01) and registered with the Chinese Clinical Trial Registry (ChiCTR2000029869). Any changes to the trial protocol will be maintained as a program addendum, and the revised protocol will be submitted to the Ethics Committee for rereview. The trained principal investigators or study coordinators will recruit the patients and conduct the process of obtaining informed consent. They will need to ensure that study participants or legal representatives receive sufficient information and time to sign the informed consent form prior to the study. Any modification of the study protocol or AEs attributable to this study will be reported to the research ethics committees.

## Discussion

The high transmission efficiency of COVID-19 has had a significantly negative impact on the global economy and community [17]. COVID-19 can cause lung inflammation and infiltration, with inflammatory cytokine storms induced by SARS-CoV-2 infection [17]. The current management strategy mainly relies on supportive therapies. Other agents, including lopinavir (LPV)-ritonavir, remdesivir, chloroquine/hydroxychloroquine and favipiravir, have been assessed in clinical trials [18]. TCM was first documented approximately 2500 years ago and then popularized by globalization. Chinese medicine has prominent value and extensive applications in the treatment of COVID-19, since no other agents have been proven to be effective [7, 19]. Additionally, TCM possesses the advantages of being simple, convenient, efficient and inexpensive with fewer side effects.

Baidu Jieduan granules, as an experiential prescription for the treatment of COVID-19, evolved from Maxing Shigan decoction and our experiential prescription, Jinhong decoction, with the addition of *Verbena officinalis L.* (*Mabiancao*), *Polygonum cuspidatum* (*Huzhang*), *Scutellariae Radix* (*Huangqin*) and *Bombyx batryticatus* (Jiangchan). *Verbena officinalis L.* and *Polygonum cuspidatum* have the effects of expelling wind and removing dampness, clearing heat and detoxifying the body, invigorating the circulation of blood, relieving cough and reducing sputum production [20, 21]. *Bombyx batryticatus* can eliminate external wind, resolve phlegm, dissipate nodules, clear heat and dissipate stagnant heat [22]. *Scutellariae Radix* can clear heat patterns, especially of the upper Jiao, dry dampness and eliminate toxicity [23]. Traditional medicine theories serve as a powerful guide in prescribing Chinese herbal formulas.

This trial is a well-designed, multicentre open-label RCT from the perspective of evidence-based medicine to assess the efficacy and safety of Baidu Jieduan granules in the management of COVID-19 for the first time. The trial can provide a clinical basis for adopting the “San Tong” and “Truncation and Reversion” strategies in the prevention and treatment of patients with COVID-19 and further enrich the theory and practice of treating infectious diseases with TCM. Nevertheless, the design of the trial also has potential limitations. It is not a double-blind, placebo-controlled clinical trial.

### Trial status

The protocol version is 1.0, 1 Feb 2020. We are currently recruiting participants from February 2020. It is estimated that up to 300 participants will be enrolled by December 30, 2020.

## Supplementary Information

**Additional file 1.** SPIRIT Checklist.

**Additional file 2.** Informed Consent Form

**Additional file 3.** Inspection Report

## Data Availability

The datasets analysed during the current study are available from the corresponding author on reasonable request.

## Abbreviations

COVID-2019: 2019 Novel coronavirus
SIRT1: Silent information regulator 1
AE: Adverse event
AEs: Adverse events
CRF: Case report form
SPIRIT: Standard protocol items recommendations for interventional trials
CT: Chest computed tomography
GCP: Good clinical practice
TCM: Traditional chinese medicine
CRO: Clinical research organization
