# Supplementary material for: Baidu Jieduan granules, traditional Chinese medicine, in the treatment of moderate coronavirus disease-2019 (COVID-19): study protocol for an open-label, randomized controlled clinical trial
Source: Trials. 2021 Jul 22;22:476. doi: 10.1186/s13063-021-05418-y (PMC8295640; doi:10.1186/s13063-021-05418-y)
Supplement: Supplementary file 1 — Additional file 1. SPIRIT Checklist. [file 13063_2021_5418_MOESM1_ESM.doc]

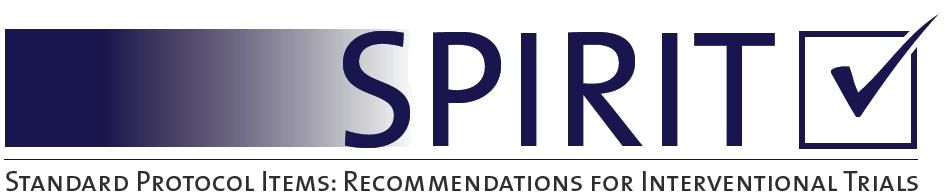


SPIRIT 2013 Checklist: Recommended items to address in a clinical trial protocol and related documents*


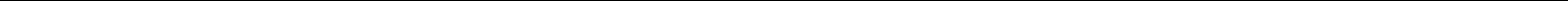


| **Section/item** | **Item** | **Description** | **Addressed on** |
| --- | --- | --- | --- |
|  | **No** |  | **page number** |
|  | |  |  |
| **Administrative information** | |  |  |
| Title | 1 | Descriptive title identifying the study design, population, interventions, and, if applicable, trial acronym | ______1_____ |
| Trial registration | 2a | Trial identifier and registry name. If not yet registered, name of intended registry | _______3____ |
|  | 2b | All items from the World Health Organization Trial Registration Data Set | _ Please refer to Item 2a and registration in the Chinese clinical trial registry, ChiCTR2000029869_ |
| Protocol version | 3 | Date and version identifier | _____15___ |
| Funding | 4 | Sources and types of financial, material, and other support | _____16____ |
| Roles and | 5a | Names, affiliations, and roles of protocol contributors | __1,17,_18_______ |
| responsibilities | 5b | Name and contact information for the trial sponsor | ______1____ |
|  |
|  | 5c | Role of study sponsor and funders, if any, in study design; collection, management, analysis, and | . |
|  |  | interpretation of data; writing of the report; and the decision to submit the report for publication, including | ___16______ |
|  |  | whether they will have ultimate authority over any of these activities |  |
|  | 5d | Composition, roles, and responsibilities of the coordinating centre, steering committee, endpoint | ______11____ |
|  |  | adjudication committee, data management team, and other individuals or groups overseeing the trial, if |  |
|  |  | applicable (see Item 21a for data monitoring committee) |  |

| **Introduction** |  |  |  |
| --- | --- | --- | --- |
| Background and | 6a | Description of research question and justification for undertaking the trial, including summary of relevant | ______3,4_____ |
| rationale |  | studies (published and unpublished) examining benefits and harms for each intervention |  |
|  | 6b | Explanation for choice of comparators | ______3,4,5__ |
| Objectives | 7 | Specific objectives or hypotheses | ____5______ |
| Trial design | 8 | Description of trial design including type of trial (eg, parallel group, crossover, factorial, single group), |  |
|  |  | allocation ratio, and framework (eg, superiority, equivalence, noninferiority, exploratory) | _____5_____ |
| **Methods: Participants, interventions, and outcomes** | | |  |
| Study setting | 9 | Description of study settings (eg, community clinic, academic hospital) and list of countries where data will | _____5,6______ |
|  |  | be collected. Reference to where list of study sites can be obtained |  |
| Eligibility criteria | 10 | Inclusion and exclusion criteria for participants. If applicable, eligibility criteria for study centres and | _____6,7_____ |
|  |  | individuals who will perform the interventions (eg, surgeons, psychotherapists) |  |
| Interventions | 11a | Interventions for each group with sufficient detail to allow replication, including how and when they will be | _____8,9___ |
|  |  | administered |  |
|  | 11b | Criteria for discontinuing or modifying allocated interventions for a given trial participant (eg, drug dose | ___7,_____ |
|  |  | change in response to harms, participant request, or improving/worsening disease) |  |
|  | 11c | Strategies to improve adherence to intervention protocols, and any procedures for monitoring adherence | ____7_______ |
|  |  | (eg, drug tablet return, laboratory tests) |  |
|  | 11d | Relevant concomitant care and interventions that are permitted or prohibited during the trial | __7,9______ |
| Outcomes | 12 | Primary, secondary, and other outcomes, including the specific measurement variable (eg, systolic blood |  |
|  |  | pressure), analysis metric (eg, change from baseline, final value, time to event), method of aggregation (eg, | ____9,10______ |
|  |  | median, proportion), and time point for each outcome. Explanation of the clinical relevance of chosen |  |
|  |  | efficacy and harm outcomes is strongly recommended |  |
| Participant timeline | 13 | Time schedule of enrolment, interventions (including any run-ins and washouts), assessments, and visits for | __10,_Figure2____ |
|  |  | participants. A schematic diagram is highly recommended (see Figure) |  |

| Sample size | 14 | Estimated number of participants needed to achieve study objectives and how it was determined, including | ___8_____ |
| --- | --- | --- | --- |
|  |  | clinical and statistical assumptions supporting any sample size calculations |  |
| Recruitment | 15 | Strategies for achieving adequate participant enrolment to reach target sample size | Patients will be recruited following admission to hospital for positive of SARS-CoV-2 nucleic acid testing ___ COVID-19 has strong infectious ability. Any patient who is identified will be isolated and treated. Till now, there is no specific drugs for COVID-19. _ The patients have high compliance. _ |

**Methods: Assignment of interventions (for controlled trials)**

| Allocation: |  |  |  |  |
| --- | --- | --- | --- | --- |
| Sequence | 16a | Method of generating the allocation sequence (eg, computer-generated random numbers), and list of any | ____8_____ | |
| generation |  | factors for stratification. To reduce predictability of a random sequence, details of any planned restriction |  |  |
|  |  | (eg, blocking) should be provided in a separate document that is unavailable to those who enrol participants |  |  |
|  |  | or assign interventions |  |  |
| Allocation | 16b | Mechanism of implementing the allocation sequence (eg, central telephone; sequentially numbered, | _____8______ | |
| concealment |  | opaque, sealed envelopes), describing any steps to conceal the sequence until interventions are assigned |  |  |
| mechanism |  |  |  |  |
| Implementation | 16c | Who will generate the allocation sequence, who will enrol participants, and who will assign participants to | ______8_ |  |
|  |  | interventions |  |  |
| Blinding (masking) | 17a | Who will be blinded after assignment to interventions (eg, trial participants, care providers, outcome | The design is open label |  |
|  |  | assessors, data analysts), and how | with only assessors being blinded |  |
|  | 17b | If blinded, circumstances under which unblinding is permissible, and procedure for revealing a participant’s | The unblinding |  |
|  |  | allocated intervention during the trial | will not occur |  |
| **Methods: Data collection, management, and analysis** | | |  |  |
| Data collection | 18a | Plans for assessment and collection of outcome, baseline, and other trial data, including any related | __12 __ | |
| methods |  | processes to promote data quality (eg, duplicate measurements, training of assessors) and a description of |  |  |
|  |  | study instruments (eg, questionnaires, laboratory tests) along with their reliability and validity, if known. |  |  |
|  |  | Reference to where data collection forms can be found, if not in the protocol |  |  |
|  | 18b | Plans to promote participant retention and complete follow-up, including list of any outcome data to be | ___7____ |  |
|  |  | collected for participants who discontinue or deviate from intervention protocols |  |  |

| Data management | 19 | Plans for data entry, coding, security, and storage, including any related processes to promote data quality | ____12_____ | |
| --- | --- | --- | --- | --- |
|  |  | (eg, double data entry; range checks for data values). Reference to where details of data management |  |  |
|  |  | procedures can be found, if not in the protocol |  |  |
| Statistical methods | 20a | Statistical methods for analysing primary and secondary outcomes. Reference to where other details of the | _10,11________ | |
|  |  | statistical analysis plan can be found, if not in the protocol |  |  |
|  | 20b | Methods for any additional analyses (eg, subgroup and adjusted analyses) | __10,11____ | |
|  | 20c | Definition of analysis population relating to protocol non-adherence (eg, as randomised analysis), and any |  |  |
|  |  | statistical methods to handle missing data (eg, multiple imputation) | _10,11______ |  |
| **Methods: Monitoring** | |  |  |  |
| Data monitoring | 21a | Composition of data monitoring committee (DMC); summary of its role and reporting structure; statement of | NA,_The Ethics Committee of Huangshi Hospital of Traditional Chinese Medicine, none of the Funders of this RCT and the provider of the intervention Beijing Tcmages Pharmaceutical Co did not consider it necessary to set up an independent Data Monitoring Committee (DMC) as the Baidu Jieduan granule was not considered to be a high risk. Thus interim analysis was not considered as there were no anticipated problems that would be detrimental to the participant______ |  |
|  |  | whether it is independent from the sponsor and competing interests; and reference to where further details |  |  |
|  |  | about its charter can be found, if not in the protocol. Alternatively, an explanation of why a DMC is not |  |  |
|  |  | needed |  |  |
|  | 21b | Description of any interim analyses and stopping guidelines, including who will have access to these interim | ____11_______ | |
|  |  | results and make the final decision to terminate the trial |  |  |
| Harms | 22 | Plans for collecting, assessing, reporting, and managing solicited and spontaneously reported adverse | ____10____ |  |
|  |  | events and other unintended effects of trial interventions or trial conduct |  |  |
| Auditing | 23 | Frequency and procedures for auditing trial conduct, if any, and whether the process will be independent | ____12, once a month _______ | |
|  |  | from investigators and the sponsor |  |  |
| **Ethics and dissemination** | |  |  |  |
| Research ethics | 24 | Plans for seeking research ethics committee/institutional review board (REC/IRB) approval | _____12____ |  |
| approval |  |  |  |  |
| Protocol | 25 | Plans for communicating important protocol modifications (eg, changes to eligibility criteria, outcomes, | _____12_______ | |
| amendments |  | analyses) to relevant parties (eg, investigators, REC/IRBs, trial participants, trial registries, journals, |  |  |
|  |  | regulators) |  |  |

| Consent or assent | 26a | Who will obtain informed consent or assent from potential trial participants or authorised surrogates, and | ____13_____ |
| --- | --- | --- | --- |
|  |  | how (see Item 32) |  |
|  | 26b | Additional consent provisions for collection and use of participant data and biological specimens in ancillary | ____NA_____ |
|  |  | studies, if applicable | The participant data and biological specimens are only intended for the clinical trial use |
| Confidentiality | 27 | How personal information about potential and enrolled participants will be collected, shared, and maintained | _____11_____ |
|  |  | in order to protect confidentiality before, during, and after the trial |  |
| Declaration of | 28 | Financial and other competing interests for principal investigators for the overall trial and each study site | ______15_____ |
| interests |  |  |  |
| Access to data | 29 | Statement of who will have access to the final trial dataset, and disclosure of contractual agreements that | ______12_ __ |
|  |  | limit such access for investigators |  |
| Ancillary and post- | 30 | Provisions, if any, for ancillary and post-trial care, and for compensation to those who suffer harm from trial | __ Informed Consent form ____ |
| trial care |  | participation |  |
| Dissemination policy | 31a | Plans for investigators and sponsor to communicate trial results to participants, healthcare professionals, | ______11_____ |
|  |  | the public, and other relevant groups (eg, via publication, reporting in results databases, or other data |  |
|  |  | sharing arrangements), including any publication restrictions |  |
|  | 31b | Authorship eligibility guidelines and any intended use of professional writers | ______10_____ |
|  | 31c | Plans, if any, for granting public access to the full protocol, participant-level dataset, and statistical code | The datasets analysed during the current study are available from the corresponding author on reasonable request |
| **Appendices** |  |  |  |
| Informed consent | 32 | Model consent form and other related documentation given to participants and authorised surrogates | _15 ____________ |
| materials |  |  |  |
|  |  |  |  |
|  |  |  |  |
| Biological | 33 | Plans for collection, laboratory evaluation, and storage of biological specimens for genetic or molecular | __NA___ |
| specimens |  | analysis in the current trial and for future use in ancillary studies, if applicable | Biological specimens may have potent infectious activity. We will not conduct genetic or molecular analysis in the current trial and for future use. |
|  |  |  |  |
