# Supplementary material for: Baidu Jieduan granules, traditional Chinese medicine, in the treatment of moderate coronavirus disease-2019 (COVID-19): study protocol for an open-label, randomized controlled clinical trial
Source: Trials. 2021 Jul 22;22:476. doi: 10.1186/s13063-021-05418-y (PMC8295640; doi:10.1186/s13063-021-05418-y)
Supplement: Supplementary file 2 — Additional file 2. Informed Consent Form [file 13063_2021_5418_MOESM2_ESM.docx]

Informed Consent Form - Information for Subjects

Dear Wardmate (family members):

You (or your family member) are currently suffering from the novel coronavirus pneumonia. Since there are no specific medications available for this virus, we invite you (or your family) to participate in a multicenter, randomized, controlled study of Chinese medicine for the treatment of severe novel coronavirus pneumonia. Before you decide whether or not to participate in this study, please read the following as carefully as possible, it will help you to understand the details of the clinical trial, the reasons for conducting the study, the procedures and duration of the research, and the possible benefits, discomfort, and risks of participating in the study. You can also discuss this with your relatives or ask your doctor for further explanation to help you decide whether to participate in this clinical study. Should you have any questions, please enquire your doctor.

1. **Background and objectives of the study:**
   1. **Research Background**

The clinical manifestations of the novel coronavirus pneumonia are fever, fatigue, and dry cough. Approximately half of the patients may experience respiratory distress, and severe cases develop acute respiratory distress syndrome rapidly, intractable metabolic acidosis, coagulation dysfunction, septic shock, multi-organ failure syndrome, and other severe complications, which have posed a high risk of death. However, there are no potent antiviral drugs currently, and clinical treatment is mainly based on conventional symptomatic treatment and life support therapy.

- 1. **Research Object**

To validate the efficacy, safety of the *Baidu Jieduan Granule* in the treatment of novel coronavirus pneumonia, and to provide evidence-based medical proof for the clinical exploration of the novel coronavirus pneumonia prevention and treatment strategies as well as the formulation of integrating Chinese and Western medicine treatment plans.

1. **Methods**

This study adopts a prospective randomized controlled study design. If you meet the inclusion criteria for this study, you may be allocated to an intervention group that combines oral *Baidu Jieduan Granule* with conventional treatment of the novel coronavirus pneumonia; or to a control group that receives conventional treatment of the novel coronavirus pneumonia only. Your participation in this study will not affect your routine treatment from the physicians.

The Baidu Jieduan Granule is composed of *Rheum palmatum L.* stem (Dahuang), *Sargentodoxa cuneata (Oliv.) Rehd. et Wils.* (Hongteng), *Taraxacum mongolicumHand.-Mazz.* (Pugongying), *Raw Gypsum* (Sheng Shigao), *Herba Ephedra* (Mahuang)，*Talcum* (Huashi), *Amygdalus Communis Vas* (Xingren), *Radix Glycyrrhizae* (Gancao), *Verbena officinalis L.*(Mabiancao), *Polygonum cuspidatum* (Huzhang), *Scutellariae Radix* (Huangqin), *Bombyx Batryticatus* (Jiangchan). The main functions are restoration and reversing the disease, clearing away heat and toxic material, promoting blood circulation and clearing the hollow viscera. If you are assigned to an intervention group, you will need to take the medication of the Prescription continuously for 14 days.

This study will record your condition and clinical information related to the disease: including medical history; routine medical tests (e.g., routine blood and urine tests, liver and kidney function tests, coagulation function tests, clinical biochemistry tests, blood gas analysis examinations, radiograph examinations, various relevant medical scores, etc.). To objectively evaluate the changes in condition, you will also be asked in detail and document your daily variation in medical condition and your prognosis on the 14th day after enrolling in the group. These therapeutic measures and medical examinations are routine items necessary for the diagnosis and treatment of the novel coronavirus pneumonia, which does not include any special tests and procedures beyond those essential for conventional diagnosis and treatment, with no additional medical costs.

1. **Responsibilities of the subjects**

Patients are required to cooperate with the requirements of the study protocol and to receive reasonable medical treatment as prescribed by the physician during the participation of the trial.

1. **Rights of the subjects**

You are participating in this study voluntarily and not obligated to participate in this study for receiving treatment of the disease. You may refuse to participate, or you may withdraw from the study at any time without any reason after enrollment of the trial. None of these decisions will be subject to discrimination or retaliation, nor will they affect your regular treatment.

You may keep yourself informed about information and research developments related to this study. If you have any questions related to this study, or if you experience any discomfort during the study, or if this study involves matters of interest to you, you may always consult with your physician. If you may wish to make a complaint while participating in this study, please contact the Ethics Committee of the hospital.

1. **Possible benefits of participating in the study**

You and the community are likely to benefit from this study. These benefits include the opportunity of improvement in your condition, the opportunity of receiving free treatment with the Baidu Jieduan Granule and the possibility of this study may help other patients with similar conditions.

Treatment and related medical examinations will be performed according to the routine protocol of this study regardless of your participation in this study. Consequently, participation in this study will not additionally increase your medical cost, and the observation and treatment of your condition will be more comprehensive and beneficial.

1. **Possible risks and discomfort in the study and risk prevention measures to be taken**

Doctors will closely monitor the development of the patient's condition during the study and will have comprehensive plans in place for the management of adverse events.

Doctors will do their best to prevent and treat the possible damage that may result from this study. In the event of any adverse event in the clinical trial, a medical expert committee will determine whether it is related to the drug in the Baidu Jieduan Granule .

The cost of treatment and corresponding financial compensation for research-related damage will be paid in accordance with the provisions of Good Clinical Practice of China

1. **Personal privacy protection**

Your name will be phonetically abbreviated in place of the various medical records in this study. Your medical records and information will be kept at the hospital and can only be accessible to the authorized researcher, research authority and ethics committee. Any public reporting of the results of this study will not disclose your identity.

You may choose not to participate in the study or withdraw from the study at any time after notifying the investigator without any discrimination or retaliation. Any medical treatment and rights you may have will not be affected as a result.

Your participation in this study is voluntary. You can keep track of the information materials related to this study. If you have questions related to this study or any injury has occurred in connection with this study, or if you have questions about the rights of subjects, you may contact the physician who is in charge.

In case of emergency, please contact the investigator: Contact Tel:

**Informed Consent Form - Consent Signature Page**

I have read the introduction of this study and had the opportunity to discuss and ask questions about this study with my doctor. All the questions I asked have been answered to my satisfaction.

I am aware of the possible risks and benefits of participating in this study and understand that participation is voluntary. I have asked for details related to the research, and all relevant questions asked to have been answered. In the meantime, I have had sufficient time to consider this with my family, and am well aware of the following:

- I can always consult my doctor for more information.
- All my personal information is confidential; my right to privacy and information will be guaranteed.
- I can withdraw from this study at any time without discrimination or retaliation; my medical treatment will not be affected.
- I consent to access to my medical records with the approval of the researcher, the research authority, and the ethics committee.
- I will be given a signed and dated copy of the informed consent form.

I decided to agree to participate in this study and to follow medical advice to the best of my ability.

Signature of subject or legal representative: Date: 2020

Relationship between the signatory and the subject: Telephone number.

I confirm that the details of this study, including the rights and possible benefits and risks, have been accurately explained to the subjects, and their questions have been answered.

Subjects who volunteered to participate in the study have been given a copy of their signed informed consent form.

Signature of the investigator: Date.

Investigator Telephone number:
