# Supplementary material for: Baidu Jieduan granules, traditional Chinese medicine, in the treatment of moderate coronavirus disease-2019 (COVID-19): study protocol for an open-label, randomized controlled clinical trial
Source: Trials. 2021 Jul 22;22:476. doi: 10.1186/s13063-021-05418-y (PMC8295640; doi:10.1186/s13063-021-05418-y)
Supplement: Supplementary file 3 — Additional file 3. Inspection Report [file 13063_2021_5418_MOESM3_ESM.pdf]

检验报告书

文件编码：SOPD1022-RD05-03

报告书编号：C201911265　　原料□净料□中间品□待包装品□成品■辅料□包材□复检□

检品名称	炒僵蚕配方颗粒	物料编码	1000000543
规　　格	250g/瓶	批　　号	19041361
本批数量	2258瓶	检品数量	1000g
检品来源		产　　地	广西壮族自治区桂林市平乐县
请验日期	2019-11-08	检验项目	全检
报告日期	2019-11-15	检验编号	C1911265
检验依据	《炒僵蚕配方颗粒质量标准》《炒僵蚕配方颗粒检验标准操作规程》		

外观性状	本品为黄绿色至黄棕色颗粒；气微，味微咸	符合规定
检查		
水分	应不得过8.0%	5.07%
粒度	应不得过13%	4.6%
溶化性	应全部溶化	符合规定
装量	应符合规定	符合规定
浸出物	应不得少于14.0%	18.2%
红外指纹图谱	应符合炒僵蚕配方颗粒图谱	符合规定
微生物限度1	需氧菌总数不得过2000cfu/g	45cfu/g
微生物限度2	霉菌和酵母菌总数不得过200cfu/g	50cfu/g
控制菌检查1	大肠埃希菌不得检出/g	未检出/g
控制菌检查2	沙门菌不得检出/10g	未检出/10g

结论：

本品依据《炒僵蚕配方颗粒质量标准》检验上述项目，结果符合规定

编制人：刘微　　复核人：孙秀　　批准人：刘照伏　　质量检验章：

北京康仁堂药业有限公司 质量检验专用章

检验报告书

文件编码：SOPD1022-RD05-03

报告书编号：C202001272　　原料☐净料☐中间品☐待包装品☐成品■辅料☐包材☐复检☐

检品名称	炒苦杏仁配方颗粒	物料编码	1000000490
规　　格	250g/瓶	批　　号	19046981
本批数量	2005瓶	检品数量	1000g
检品来源		产　　地	河北省承德市隆化县
请验日期	2020-01-15	检验项目	全检
报告日期	2020-01-20	检验编号	C2001272
检验依据	《炒苦杏仁配方颗粒质量标准》《炒苦杏仁配方颗粒检验标准操作规程》		

外观性状	本品为淡黄色至黄色颗粒；无臭，味苦	符合规定
鉴别		
薄层鉴别	供试品主斑点的位置和颜色应与苦杏仁苷对照品相同	符合规定
检查		
水分	应不得过8.0%	3.35%
粒度	应不得过13%	3.8%
溶化性	应全部溶化	符合规定
装量	应符合规定	符合规定
含量测定	本品按干燥品计算，含苦杏仁苷不得少于3.0%	19%
红外指纹图谱	应符合炒苦杏仁配方颗粒图谱	符合规定
微生物限度1	需氧菌总数不得过2000cfu/g	30cfu/g
微生物限度2	霉菌和酵母菌总数不得过200cfu/g	<10cfu/g
控制菌检查	大肠埃希菌不得检出/g	未检出/g

结论：

本品依据《炒苦杏仁配方颗粒质量标准》检验上述项目，结果符合规定

编制人：王佳梅　　复核人：孙秀　　批准人：刘照伏　　质量检验章：北京康仁堂药业有限公司 质量检验专用章

检验报告书

文件编码：SOPD1022-RD05-03

报告书编号：C201910363　　原料☐净料☐中间品☐待包装品☐成品■辅料☐包材☐复检☐

检品名称	大黄配方颗粒	物料编码	1000000443
规　　格	250g/瓶	批　　号	19033371
本批数量	3383瓶	检品数量	1000g
检品来源		产　　地	四川省广元市旺苍县
请验日期	2019-10-21	检验项目	全检
报告日期	2019-10-28	检验编号	C1910363
检验依据	《大黄配方颗粒质量标准》《大黄配方颗粒检验标准操作规程》		

外观性状	本品为棕黄色至棕色颗粒；气清香，味苦而微涩	符合规定
鉴别		
薄层鉴别	供试品主斑点的位置和颜色应与大黄对照药材，芦荟大黄素、大黄酸、大黄素、大黄酚、大黄素甲醚对照品相同	符合规定
检查		
水分	应不得过8.0%	5.36%
粒度	应不得过13%	5.1%
溶化性	应全部溶化	符合规定
装量	应符合规定	符合规定
土大黄苷	应不得检出	符合规定
含量测定	本品按干燥品计算，每1g含芦荟大黄素、大黄酸、大黄素、大黄酚和大黄素甲醚的总量不得少于7.5mg	13mg/g
红外指纹图谱	应符合大黄配方颗粒图谱	符合规定
微生物限度1	需氧菌总数不得过2000cfu/g	<10cfu/g
微生物限度2	霉菌和酵母菌总数不得过200cfu/g	<10cfu/g
控制菌检查	大肠埃希菌不得检出/g	未检出/g

结论：

本品依据《大黄配方颗粒质量标准》检验上述项目，结果符合规定

编制人：刘微　　复核人：孙秀　　批准人：安君　　质量检验章：北京康仁堂药业有限公司 质量检验专用章

检验报告书

文件编码：SOPD1022-RD05-03

报告书编号：C201909178　　原料☐净料☐中间品☐待包装品☐成品■辅料☐包材☐复检☐

检品名称	大血藤配方颗粒	物料编码	1000000388
规　　格	100g/瓶	批　　号	19033991
本批数量	2380瓶	检品数量	400g
检品来源		产　　地	江西省宜春市丰城市
请验日期	2019-09-11	检验项目	全检
报告日期	2019-09-29	检验编号	C1909178
检验依据	《大血藤配方颗粒质量标准》《 大血藤配方颗粒检验标准操作规程》		

外观性状	本品为黄棕色至红棕色颗粒；气微，味苦、平	符合规定
鉴别		
薄层鉴别	供试品主斑点的位置和颜色应与大血藤对照药材相同	符合规定
检查		
水分	应不得过8.0%	4.02%
粒度	应不得过13%	4.7%
溶化性	应全部溶化	符合规定
装量	应符合规定	符合规定
浸出物	应不得少于26.0%	36.2%
红外指纹图谱	应符合大血藤配方颗粒图谱	符合规定
微生物限度1	需氧菌总数不得过2000cfu/g	<10cfu/g
微生物限度2	霉菌和酵母菌总数不得过200cfu/g	<10cfu/g
控制菌检查	大肠埃希菌不得检出/g	未检出/g

结论：

本品依据《大血藤配方颗粒质量标准》检验上述项目，结果符合规定

编制人：王佳梅　　复核人：孙秀　　批准人：安君　　质量检验章：北京康仁堂药业有限公司 质量检验专用章

检验报告书

文件编码：SOPD1022-RD05-03

报告书编号：C202001315　　原料□净料□中间品□待包装品□成品■辅料□包材□复检□

检品名称	虎杖配方颗粒	物料编码	1000000062
规　　格	100g/瓶	批　　号	20000761
本批数量	4943瓶	检品数量	400g
检品来源		产　　地	湖北省襄阳市襄城区
请验日期	2020-01-16	检验项目	全检
报告日期	2020-01-22	检验编号	C2001315
检验依据	《虎杖配方颗粒质量标准》《虎杖配方颗粒检验标准操作规程》		

外观性状	本品为棕黄色至棕褐色颗粒；气微，味微苦、涩	符合规定
鉴别		
薄层鉴别	供试品主斑点的位置和颜色应与虎杖对照药材，大黄素、大黄素甲醚对照品相同	符合规定
检查		
水分	应不得过8.0%	4.74%
粒度	应不得过13%	4.7%
溶化性	应全部溶化	符合规定
装量	应符合规定	符合规定
含量测定1	本品按干燥品计算，含大黄素不得少于0.60%	1.6%
含量测定2	本品按干燥品计算，含虎杖苷不得少于1.00%	3.89%
红外指纹图谱	应符合虎杖配方颗粒图谱	符合规定
微生物限度1	需氧菌总数不得过2000cfu/g	<10cfu/g
微生物限度2	霉菌和酵母菌总数不得过200cfu/g	<10cfu/g
控制菌检查	大肠埃希菌不得检出/g	未检出/g

结论：

本品依据《虎杖配方颗粒质量标准》检验上述项目，结果符合规定

编制人：王佳梅　　复核人：孙秀　　批准人：刘照伏　　质量检验章：北京康仁堂药业有限公司 质量检验专用章

检验报告书

文件编码：SOPD1022-RD05-03

报告书编号：C201910335　　原料☐净料☐中间品☐待包装品☐成品■辅料☐包材☐复检☐

检品名称	滑石配方颗粒	物料编码	1000000594
规　　格	100g/瓶	批　　号	19036451
本批数量	6767瓶	检品数量	400g
检品来源		产　　地	广西壮族自治区桂林市龙胜各族自治县
请验日期	2019-10-19	检验项目	全检
报告日期	2019-10-30	检验编号	C1910335
检验依据	《滑石配方颗粒质量标准》《滑石配方颗粒检验标准操作规程》		

外观性状	本品为白色至黄白色颗粒；气微，无味	符合规定
检查		
水分	应不得过6.0%	2.2%
粒度	应不得过13%	1.2%
溶化性	应混悬均匀	符合规定
装量	应符合规定	符合规定
微生物限度1	需氧菌总数不得过20000cfu/g	<10cfu/g
微生物限度2	霉菌和酵母菌总数不得过200cfu/g	<10cfu/g
控制菌检查1	大肠埃希菌不得检出/g	未检出/g
控制菌检查2	沙门菌不得检出/10g	未检出/10g
控制菌检查3	耐胆盐革兰阴性菌应小于100cfu/g	<10cfu/g

结论：

本品依据《滑石配方颗粒质量标准》检验上述项目，结果符合规定

编制人：刘微　　复核人：孙秀　　批准人：安君　　质量检验章：北京康仁堂药业有限公司 质量检验专用章

检验报告书

文件编码：SOPD1022-RD05-03

报告书编号：C202001404　　原料☐净料☐中间品☐待包装品☐成品■辅料☐包材☐复检☐

检品名称	黄芩配方颗粒	物料编码	1000000460
规　　格	250g/瓶	批　　号	19046901
本批数量	3770瓶	检品数量	1000g
检品来源		产　　地	山西省临汾市襄汾县
请验日期	2020-01-20	检验项目	全检
报告日期	2020-01-30	检验编号	C2001404
检验依据	《黄芩配方颗粒质量标准》《黄芩配方颗粒检验标准操作规程》		

外观性状	本品为黄色至黄棕色的颗粒；气微，味苦	符合规定
鉴别		
薄层鉴别	供试品主斑点的位置和颜色应与黄芩对照药材，黄芩苷对照品、黄芩素对照品、汉黄芩素对照品相同	符合规定
检查		
水分	应不得过6.0%	3.3%
粒度	应不得过13%	4.7%
溶化性	应全部溶化	符合规定
装量	应符合规定	符合规定
含量测定	本品按干燥品计算，每1g含黄芩苷不得少于150.0mg	164.9mg/g
浸出物	应不得少于20.0%	29.4%
红外指纹图谱	应符合黄芩配方颗粒图谱	符合规定
微生物限度1	需氧菌总数不得过2000cfu/g	<10cfu/g
微生物限度2	霉菌和酵母菌总数不得过200cfu/g	<10cfu/g
控制菌检查	大肠埃希菌不得检出/g	未检出/g

结论：

本品依据《黄芩配方颗粒质量标准》检验上述项目，结果符合规定

编制人：王佳梅　　复核人：孙秀　　批准人：刘照伏　　质量检验章：北京康仁堂药业有限公司 质量检验专用章

检验报告书

文件编码：SOPD1022-RD05-03

报告书编号：C201907344　　原料☐净料☐中间品☐待包装品☐成品■辅料☐包材☐复检☐

检品名称	麻黄配方颗粒	物料编码	1000000280
规　　格	100g/瓶	批　　号	19024311
本批数量	6872瓶	检品数量	400g
检品来源		产　　地	内蒙古自治区鄂尔多斯市鄂托克前旗
请验日期	2019-07-19	检验项目	全检
报告日期	2019-07-26	检验编号	C1907344
检验依据	《麻黄配方颗粒质量标准》《麻黄配方颗粒检验标准操作规程》		

外观性状	本品为黄色至棕黄色颗粒；气微香，味涩、微苦	符合规定
鉴别		
理化鉴别	应符合规定	符合规定
薄层鉴别	供试品主斑点的位置和颜色应与麻黄对照药材，盐酸麻黄碱对照品相同	符合规定
检查		
水分	应不得过6.0%	4.5%
粒度	应不得过13%	3.4%
溶化性	应全部溶化	符合规定
装量	应符合规定	符合规定
含量测定	本品按干燥品计算，含盐酸麻黄碱和盐酸伪麻黄碱的总量不得少于1.5%	4.9%
红外指纹图谱	应符合麻黄配方颗粒图谱	符合规定
微生物限度1	需氧菌总数不得过2000cfu/g	250cfu/g
微生物限度2	霉菌和酵母菌总数不得过200cfu/g	100cfu/g
控制菌检查	大肠埃希菌不得检出/g	未检出/g

结论：

本品依据《麻黄配方颗粒质量标准》检验上述项目，结果符合规定

编制人：刘微　　复核人：孙秀　　批准人：周坤　　质量检验章：北京康仁堂药业有限公司 质量检验专用章

检验报告书

文件编码：SOPD1022-RD05-03

报告书编号：C201906388　　原料□净料□中间品□待包装品□成品■辅料□包材□复检□

检品名称	马鞭草配方颗粒	物料编码	1000000282
规　　格	100g/瓶	批　　号	19021101
本批数量	2520瓶	检品数量	400g
检品来源		产　　地	贵州省毕节市金沙县
请验日期	2019-06-22	检验项目	全检
报告日期	2019-06-28	检验编号	C1906388
检验依据	《马鞭草配方颗粒质量标准》《马鞭草配方颗粒检验标准操作规程》		

外观性状	本品为浅黄色至黄棕色颗粒；气微，味苦	符合规定
鉴别		
薄层鉴别	供试品主斑点的位置和颜色应与马鞭草对照药材，熊果酸对照品相同	符合规定
检查		
水分	应不得过6.0%	4.3%
粒度	应不得过13%	4.7%
溶化性	应全部溶化	符合规定
装量	应符合规定	符合规定
浸出物	应不得少于20.0%	36.7%
红外指纹图谱	应符合马鞭草配方颗粒图谱	符合规定
微生物限度1	需氧菌总数不得过2000cfu/g	<10cfu/g
微生物限度2	霉菌和酵母菌总数不得过200cfu/g	<10cfu/g
控制菌检查	大肠埃希菌不得检出/g	未检出/g

结论：

本品依据《马鞭草配方颗粒质量标准》检验上述项目，结果符合规定

编制人：刘微　　复核人：孙秀　　批准人：安君　　质量检验章：北京康仁堂药业有限公司 质量检验专用章

检验报告书

文件编码：SOPD1022-RD05-03

报告书编号：C201912715　　原料☐净料☐中间品☐待包装品☐成品■辅料☐包材☐复检☐

检品名称	蒲公英配方颗粒	物料编码	1000000512
规　　格	250g/瓶	批　　号	19047721
本批数量	3427瓶	检品数量	1000g
检品来源		产　　地	甘肃省定西市陇西县
请验日期	2019-12-28	检验项目	全检
报告日期	2020-01-02	检验编号	C1912715
检验依据	《蒲公英配方颗粒质量标准》《蒲公英配方颗粒检验标准操作规程》		

外观性状	本品为浅棕黄色至黄棕色的颗粒，气微，味苦	符合规定
鉴别		
薄层鉴别	供试品主斑点的位置和颜色应与蒲公英对照药材、咖啡酸对照品相同	符合规定
检查		
水分	应不得过8.0%	6.00%
粒度	应不得过13%	3.2%
溶化性	应全部溶化	符合规定
装量	应符合规定	符合规定
含量测定	本品按干燥品计算，每1g含咖啡酸不得少于0.4mg	0.8mg/g
浸出物	应不得少于20.0%	26.5%
红外指纹图谱	应符合蒲公英配方颗粒图谱	符合规定
微生物限度1	需氧菌总数不得过2000cfu/g	<10cfu/g
微生物限度2	霉菌和酵母菌总数不得过200cfu/g	10cfu/g
控制菌检查	大肠埃希菌不得检出/g	未检出/g

结论：

本品依据《蒲公英配方颗粒质量标准》检验上述项目，结果符合规定

编制人：李雪婷　　复核人：孙秀　　　批准人：刘照伏　　　质量检验章：北京康仁堂药业有限公司 质量检验专用章

检验报告书

文件编码：SOPD1022-RD05-03

报告书编号：C201910385　　原料☐净料☐中间品☐待包装品☐成品■辅料☐包材☐复检☐

检品名称	生甘草配方颗粒	物料编码	1000000452
规　　格	250g/瓶	批　　号	19033231
本批数量	3614瓶	检品数量	1000g
检品来源		产　　地	甘肃省定西市陇西县
请验日期	2019-10-21	检验项目	全检
报告日期	2019-10-31	检验编号	C1910385
检验依据	《生甘草配方颗粒质量标准》《生甘草配方颗粒检验标准操作规程》		

外观性状	本品为黄色至黄棕色的颗粒；气微，味甜而特异	符合规定
鉴别		
薄层鉴别	供试品主斑点的位置和颜色应与甘草对照药材、甘草酸铵对照品相同	符合规定
检查		
水分	应不得过8.0%	3.21%
粒度	应不得过13%	6.9%
溶化性	应全部溶化	符合规定
装量	应符合规定	符合规定
重金属及有害元素	应符合规定	符合规定
有机氯农药残留量	应符合规定	符合规定
含量测定1	本品按干燥品计算，每1g含甘草苷不得少于8.0mg	15.7mg/g
含量测定2	本品按干燥品计算，每1g含甘草酸不得少于34.8mg	77.4mg/g
红外指纹图谱	应符合生甘草配方颗粒图谱	符合规定
微生物限度1	需氧菌总数不得过2000cfu/g	<10cfu/g
微生物限度2	霉菌和酵母菌总数不得过200cfu/g	<10cfu/g
控制菌检查	大肠埃希菌不得检出/g	未检出/g

结论：

本品依据《生甘草配方颗粒质量标准》检验上述项目，结果符合规定

编制人：刘微　　复核人：孙秀　　批准人：安君　　质量检验章：北京康仁堂药业有限公司 质量检验专用章

检验报告书

文件编码：SOPD1022-RD05-03

报告书编号：C202001033　　原料□净料□中间品□待包装品□成品■辅料□包材□复检□

检品名称	生石膏配方颗粒	物料编码	1000000545
规　　格	250g/瓶	批　　号	19047961
本批数量	3024瓶	检品数量	1000g
检品来源		产　　地	山西省运城市闻喜县
请验日期	2020-01-04	检验项目	全检
报告日期	2020-01-09	检验编号	C2001033
检验依据	《生石膏配方颗粒质量标准》《生石膏配方颗粒检验标准操作规程》		

外观性状	本品为白色至灰白色颗粒；气微，味淡	符合规定
鉴别		
理化鉴别	应符合规定	符合规定
检查		
水分	应不得过8.0%	6.23%
粒度	应不得过13%	2.9%
溶化性	应混悬均匀	符合规定
装量	应符合规定	符合规定
含量测定	本品按干燥品计算，含含水硫酸钙不得少于16.0%	21.5%
微生物限度1	需氧菌总数不得过20000cfu/g	<10cfu/g
微生物限度2	霉菌和酵母菌总数不得过200cfu/g	10cfu/g
控制菌检查1	大肠埃希菌不得检出/g	未检出/g
控制菌检查2	沙门菌不得检出/10g	未检出/10g
控制菌检查3	耐胆盐革兰阴性菌应小于100cfu/g	<10cfu/g

结论：

本品依据《生石膏配方颗粒质量标准》检验上述项目，结果符合规定

编制人：李雪婷　　复核人：孙秀　　批准人：刘照伏　　质量检验章：北京康仁堂药业有限公司 质量检验专用章
